# Temporal trend of measles cases and impact of vaccination on mortality in Jigawa State, Nigeria, 2013-2017: a secondary data analysis

**DOI:** 10.11604/pamj.supp.2020.35.1.19780

**Published:** 2020-02-19

**Authors:** Aisha Sani Faruk, Ayo Stephen Adebowale, Muhammad Shakir Balogun, Lydia Taiwo, Olawunmi Adeoye, Samaila Mamuda, Ndadilnasiya Endie Waziri

**Affiliations:** 1 Nigeria Field Epidemiology and Laboratory Training Program, Nigeria; 2 Department of Epidemiology and Medical Statistics, University of Ibadan, Nigeria; 3 African Field Epidemiology Network, Nigeria; 4 Nigeria Center for Disease Control; 5 Jigawa State Ministry of Health, Nigeria

**Keywords:** Measles, vaccination, Nigeria

## Abstract

**Introduction:**

Measles is a highly infectious vaccine-preventable viral disease that mostly affects children less than five years old. Jigawa located in the north-west zone has the highest burden of measles in Nigeria. We reviewed Jigawa State measles surveillance data to identify measles trend and factors associated with mortality.

**Methods:**

We conducted a secondary data analysis of measles specific integrated disease surveillance and response data for Jigawa State from January 2013 to December 2017. We extracted relevant variables and analyzed data using descriptive statistics and logistic regression model (α = 0.05). We estimated seasonal variation using an additive time series model.

**Results:**

A total of 6,214 cases were recorded with 1038 (16.7%) confirmed by laboratory investigation. Only 1,185 (19.7%) had at least one dose of measles vaccine. Age specific attack and fatality rates were highest among children under the age of five years (503/100,000 and 1.8% respectively). The trend showed a decrease in number of cases across all the years. Seasonal variation existed with cases peaking in the first quarter. The likelihood of mortality associated with measles was higher among cases who had no vaccination (AOR = 4.7, 95% CI: 2.9-7.5) than those who had at least one dose of measles vaccine.

**Conclusion:**

There was a decrease in the trend of measles cases, however, the vaccination coverage was very low in Jigawa State. Receiving at least one dose of measles vaccine reduces mortality among the cases. Strengthening routine immunization will reduce number of cases and mortality associated with the disease.

## Introduction

Measles is a highly infectious vaccine-preventable viral disease characterized by a prodrome of fever, cough, coryza and conjunctivitis, followed by a maculopapular rash. The disease remains one of the leading causes of death among young children, despite the availability of a safe and effective vaccine [1]. In 2017, an estimated 110,000 measles deaths occurred globally, mostly among children under the age of five. Routine measles vaccination for children, combined with mass immunization campaigns, case-based surveillance and standard case management are key public health strategies to reduce global measles deaths [1, 2]. Measles is still common in many developing countries - particularly in parts of Africa and Asia [1]. In these countries, measles case fatality rate is estimated to be 3-5% but may reach 10-30% in cases with complications [3]. Malnutrition, poor case management, complications like pneumonia, age at infection, overcrowding and underlying immune deficiency disorders are associated with the high measles mortality rate [1, 4]. High prevalence of childhood diseases including measles constitute a challenge to mortality reduction agenda in Nigeria where the under-five mortality rate is 120/1,000 live births [5]. The burden of measles is highest in the north-western region of the country with recurrent outbreaks occurring at irregular intervals [6]. Unvaccinated children are at higher risk of the disease and its complication and Nigeria has the highest number of unvaccinated children globally [1, 7]. The WHO African Region adopted a regional measles mortality reduction goal in 2001. The recommended strategies to achieve the program goal included improved case management, achieving and maintaining ≥ 80% coverage with routine measles vaccination of infants, providing a second dose of measles vaccination through supplemental immunization activities (SIAs) and intensified measles case-based surveillance [8]. In 2011, the Member States of the WHO African Region established a goal to achieve measles elimination by 2020 with the following targets: ≥95% coverage with the first dose of measles-containing vaccine (MCV1) at national and district levels, ≥ 95 SIA coverage in every district, and confirmed measles incidence of < 1 per million population in all countries, and attaining the targets for the two principal surveillance performance monitoring indicators which are: ≥ 80% of districts with ≥ 1 suspected measles case with blood specimen reported per year and a non-measles febrile rash illness rate of ≥ 2 per 100,000 population [9]. With the implementation of these recommended strategies, the African Region of the WHO has achieved 85% reduction in estimated measles deaths by the end of 2015 as compared to mortality estimates in 2000 [10]. Accelerated measles control activities started in Nigeria in 2006 with the conduct of the first catch-up measles campaign. Since then, nationwide mass vaccination campaigns were conducted every two years in the country targeting children aged 9-59 months. Consequently, the national measles vaccination coverage increased from 33% in 2006 to 42% in 2017 [5] and a significant decline in measles incidence was observed following the initial measles catch-up campaign, but later the country experienced resurgence [11]. Contribution to high prevalence of measles cases varied widely across the 36 states in Nigeria including the Federal Capital Territory. Jigawa State is among the states with highest burden of measles in Nigeria which can be attributed to the very low measles vaccination coverage of 10.4% [5]. Studies have shown that a 95% measles vaccination coverage is required to interrupt measles transmission [2, 12]. Consequently, this low coverage drives recurrent outbreaks of measles in the state. Thus, analysis of the measles surveillance data might generate information that will help in prevention and control of the disease. We therefore conducted this analysis to determine the magnitude of measles in Jigawa State, identify its trend and determine the factors associated with mortality.

## Methods

### Study design

This study is a cross-sectional study of Jigawa State measles specific Integrated Disease Surveillance and Response (IDRS) data from 2013 to 2017.

### Study setting

Jigawa State is in the north-western part of Nigeria and has twenty-seven local government areas. It lies between latitude 11°N and 13°N and longitude 8°E and 10.15°E and shares common national boundaries with Kano to the west, Katsina to the north, Yobe to the east and Bauchi to the south-east and an international border with Niger republic. It covers an area of 22,410sq.km with a population of 5,624,614 of which 20.0% are children aged 5 years and below (2015 projected figure from 2006 census). There are two seasons in a year, namely; rainy and dry seasons. The rainy season starts from April to October while the dry season covers the period of November to March. Measles transmission occurs throughout the year but peaks in the dry season.

### Data source

IDSR weekly epidemiological data for the year 2013 to 2017 were obtained from Epidemiology Unit of Jigawa State Ministry of Health. The base population figures for the estimation of attack rates were obtained through the projection of 2006 census figures for Jigawa state using the 2.9% annual growth rate.

#### Measles surveillance

Measles surveillance in Jigawa State is based on the IDSR strategy which is a reporting platform for all priority diseases. A suspected case of measles is any person with fever and maculopapular (non-vesicular) generalized rash and cough, coryza or conjunctivitis or any person in whom a clinician suspects measles. For every suspected measles case, a case investigation form was completed, and a blood specimen collected and sent to the national reference laboratory for testing for measles-specific immunoglobulin M (IgM) antibody. The designated local government disease surveillance and notification officer is responsible for the completion of the form and transportation of the specimen. Suspected measles cases are confirmed by laboratory testing, epidemiologic linkage to a confirmed case, or by clinical criteria. A laboratory confirmed case of measles was a suspected case with serological confirmation of measles specific IgM antibody in a person who had not received measles vaccination within 30 days before the specimen collection. Epidemiologically linked case was a suspected case from whom blood specimen was not collected and is linked in person, place and time to a laboratory confirmed case. While a measles associated death is defined as any death from illness in a confirmed case of measles within 1month after the onset of rash. Completed individual case investigation forms and laboratory results were entered into an Excel database. Information flows from the health facilities, through the ward focal persons to the local government disease surveillance and notification officers (DSNOs), to the state DSNO and State Epidemiologist, and then collated by the Nigeria Center for Disease Control (NCDC). Feedback goes through the opposite direction.

### Data management

Relevant data variables were sorted, extracted, and cleaned from the IDSR line list. This included age, sex, location, number of cases, date of onset of rash, vaccine doses, laboratory results and outcome. The outcome variable was disease outcome (alive/dead) while the explanatory variables were age, sex, location and vaccination status. Data were analyzed using Microsoft Excel 2016 and Epi-Info7. Frequencies and proportions were used to summarize the data while multivariate analysis was used to examine the relationship between the explanatory variables and disease outcome. The monthly reported cases of measles in a specific year was grouped into 3 months. The data were aggregated in 3-months as 1st quarter (January to March), 2nd quarter (April-June), 3rd quarter (July-September), 4th quarter (November-December) from 2013 to 2017. We decomposed the data and used the estimates of the quarters to describe the time series. This was done by computing the 3-quarter moving average in order to eliminate seasonal variations and irregular variations from the data. The number of cases in a quarter was represented by Yt and the trend line (Tt) was obtained using the seasonal variation method [13]:

$${\text{T}}_{\text{t}}=\frac{Y_{t-1}+Y_{t}+Y_{t+1}}{3}$$

In order to obtain the seasonal variation in the data, the multiplicative model was used based on the pattern exhibited by the observed data, and this is given by [13]:

$$\text{Seasonal variation (SV)}=\frac{Y_{t}}{T_{t}}*100$$

The data was deseasonalized to obtain the variation in each quarter of the year as [13]:

$$\text{Quarterly variation }({\text{QV}}_{\text{i}})=\frac{\sum_{i=1}^{L}SV_{t}}{L}-\Delta SV\left(\frac{\sum_{i=1}^{n-m}SV}{4}\right)$$

**Figure 1:**
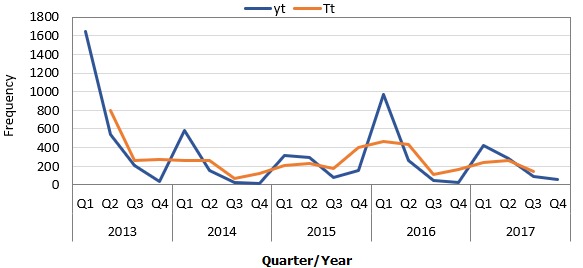
Frequency distribution of quarterly reported cases (yt) of measles and the trend line, Jigawa State, Nigeria, 2013-2017

Where ΔSV is the excess of the sum of all the seasonal variations and L is the number of quarters that are present in the seasonality of a given year. Table 1 shows the procedures involved in the estimation of seasonal variation. In Figure 1, the monthly pattern was merged into quarter on yearly basis and smoothed using a time series approach to obtain the trend.

**Table 1:** Estimation of quarterly trend of measles cases in Jigawa State, 2013-2017

Year	Quarter	Actual (Yt)	3-Qtr Moving Total	Trend (Tt)	Seasonal Variation SV=(Yt/Tt)*100
2013	Q1	1646	-	-	-
	Q2	545	2400	800.00	68.13
	Q3	209	788	262.67	79.57
	Q4	34	826	275.33	12.35
2014	Q1	583	775	258.33	225.68
	Q2	158	770	256.67	61.56
	Q3	29	204	68.00	42.65
	Q4	17	365	121.67	13.97
2015	Q1	319	630	210.00	151.90
	Q2	294	688	229.33	128.20
	Q3	75	529	176.33	42.53
	Q4	160	1209	403.00	39.70
2016	Q1	974	1399	466.33	208.86
	Q2	265	1285	428.33	61.87
	Q3	46	333	111.00	41.44
	Q4	22	490	163.33	13.47
2017	Q1	422	722	240.67	175.35
	Q2	278	785	261.67	106.24
	Q3	85	416	138.67	61.30
	Q4	53	-	-	

### Ethical consideration

Approval to use the surveillance data was sought from and granted by Public Health Department of the Jigawa State Ministry of Health Ministry. To protect patient confidentiality, personal information was de-identified during extraction and data analysis.

## Results

**Figure 2:**
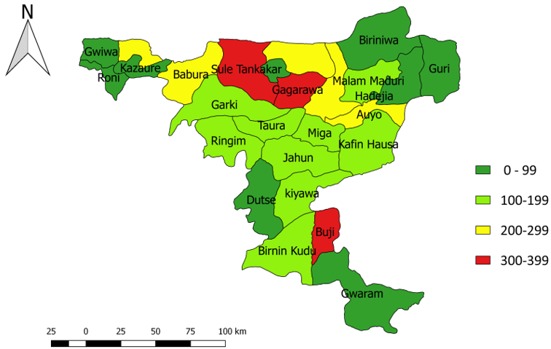
Measles attack rate by local government areas in Jigawa State, 2013 to 2017

There were 3,247 (52.3%) males and the most affected age group was 1-5 years (73.3%). Only 1,190 (19.2%) had at least one dose of measles vaccine (Table 2). Buji (284/100,000) local government area had the highest attack rate while Gumel (16/100,000) had the least attack rate reported for the five-year period (Figure 2). The overall case fatality rate (CFR) was 1.7%. The age-specific attack and case fatality rates (50.3/10,000 and 1.8% respectively) were highest among those less than five years (Table 3).

**Table 2:** Distribution of measles cases by selected demographic and health characteristics in Jigawa State, 2013-2017

Characteristics	Frequency (n= 6214)	Percentage (%)
**Age group**		
<5	4556	73.3
5-9	1469	23.6
10-14	132	2.1
15-19	38	0.6
≥20	19	0.3
**Sex**		
Male	3247	52.3
Female	2967	47.7
**Vaccination status**		
None	4974	80.0
Had at least one dose	1190	19.2
Unknown	50	0.8
**Outcome status**		
Alive	5936	95.5
Dead	106	1.7
Unknown	172	2.8

**Table 3:** Age specific attack and case fatality rates of measles cases in Jigawa State, 2013-2017

Age group (Years)	Case (%)	Deaths (%)	ASCFR^*^	Estimated age group pop^†^	ASAR^#^ /10,000 pop^†^
<5	4556 (73.4)	84 (79.3)	1.8	905597	50.3
5-9	1469 (23.6)	21 (19.8)	1.4	802035	18.3
10-14	132 (2.1)	1 (0.9)	0.8	646694	2.00
15-19	38 (0.6)	0	-	597165	0.60
≥20	19 (0.3)	0	-	2676828	0.01
**Total**	6214 (100)	106 (100)	1.7	5628319	11.0

† population;

# age specific attack rate;

* age-specific case fatality rate

**Figure 3:**
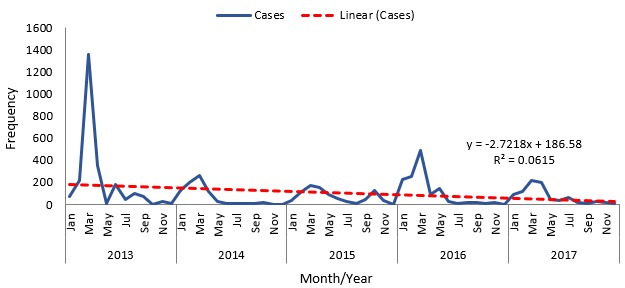
Trend of measles cases in Jigawa State, 2013-2017

A downward trend of the measles cases was observed throughout the years. There was a slight variation in the cases with only 6.2% of the variation being explained by month (Figure 3). In Table 4, the data show the adjusted seasonal variation to establish the exact variation. The data indicates that the highest cases of measles were observed in the first quarter of the year and this falls consistently through the remaining quarters of the year. The seasonal variation was found to be highest in the first quarter across all the years and fells consistently in the subsequent quarters. The adjusted seasonal variation was 1.8, 0.7, 0.4 and 0.1 for the 1st, 2nd, 3rd and 4th quarter respectively. Compared to those aged 5 years and above, those less than 5 years were more likely to die of measles (AOR= 2.0, 95% CI: 1.1-3.6). Similarly, males were more likely to die compared to their female counterparts (AOR= 1.7, 95% CI: 1.1-2.7). Also, those who were never vaccinated were more likely to die compared to those who had had at least one dose of the measles vaccine (AOR = 4.7, 95% CI: 2.9-7.5) (Table 5).

**Table 4:** Deseasonalization of seasonal variation of measles cases and estimation of quarterly variation in Jigawa State, 2013-2017

Year	Quarter					
	1	2	3	4		
2013	-	68.1	79.6	12.4		
2014	225.7	61.6	42.7	14.0		
2015	151.9	128.2	42.5	39.7		
2016	208.9	61.9	41.4	13.5		
2017	175.4	106.2	61.3	-		
**Total**	761.8	426.0	267.5	79.5		
**Average**	**190.448**	**85.2**	**53.5**	**19.9**	349.0	12.3
	12.3	12.3	12.3	12.3		
**Estimated quarterly variation**	**1.8**	**0.7**	**0.4**	**0.1**		

**Table 5:** Factors associated with measles mortality in Jigawa State, 2013-2017

Exposure factors	Dead (%) (n=86)	Alive (%) (n=5959)	OR (95% CI)	AOR (95% CI)
**Age**				
<5 years	72 (1.6)	4386 (98.4)	1.8 (1.0-3.3)	
≥5 years	14 (0.9)	1573 (99.1)		2.0 (1.1-3.6)*
**Sex**				
Male	56 (1.8)	3109 (98.2)	1.7 (1.1-2.7)	
Female		2850 (99.0)		1.7 (1.1-2.7)*
**Vaccination status**	30 (1.0)			
Unvaccinated	62 (2.8)	2160 (97.2)	4.5 (2.8-7.3)	
Vaccinated	24 (0.6)	3799 (99.4)		4.7 (2.9-7.5)*

* Significant at 5.0%

## Discussion

Our study found that the overall CFR found in Jigawa State was lower than the country’s CFR of 0.6% [6]. The age-specific fatality rate was higher among children under the age of five years with no mortality recorded in those aged 15 years and above. Likewise, the age-specific attack rate was highest among those under five children and decreased as age advances, with the lowest rate recorded among those twenty years and above. This might be explained by the lifetime immunity conferred by measles. Most of the individuals aged 5 years and above might have been exposed to the antigen either through vaccination or measles infection before that age and have acquired immunity [14]. Sule Tankarkar, Gagarawa, Kaugama, Yankwashi and Buji LGAs had the highest measles attack rates within the five-year period under review. These LGAs are border towns with hard-to-reach settlements and majority of the inhabitants are herds men who are seasonal migrants. This might explain the low immunization coverage in the LGAs. High immunization coverage and low measles attack rate found in Dutse, Kazaure, Hadejia and Gumel LGAs could be attributed to the metropolitan nature of the LGAs and emirates being situated in the area since these traditional institutions play a major role in immunization uptake. The other LGAs with attack rates of less than 100/100,000 are contiguous to these LGAs and have high immunization coverages.

The National Measles Surveillance and Outbreak Response Guidelines specified all suspected measles cases are to be confirmed by laboratory testing and thus underestimates the burden of the disease in the state. However, our study revealed that only 16.7% of all the reported measles cases were tested. This reflects that laboratory confirmation of measles is very low in Jigawa State. Similar findings were reported in Nigeria, Uganda and Ethiopia [6, 15–17]. The finding of low vaccination coverage among cases was consistent with studies in other parts of the WHO Africa region which revealed low vaccination coverage among measles cases [6, 18–20].

Our study revealed high number of measles cases in the state in 2013 following which there was a supplemental immunization activity (SIA), hence the fall in the number of cases in 2014. Ironically, there was another SIA conducted in 2015, however, there was increase in the number of measles cases in 2016. This might be due to vaccine failure or failure to achieve herd immunity. Similar findings were reported in other parts of Nigeria, Kenya and Congo where outbreaks occurred due to suboptimal measles vaccination coverage [19, 21–23]. Our model depicted a decreasing trend over the years which is in line with the goal of measles mortality reduction globally. Similar finding was reported in the country [6]. However, a contrasting finding which shows an upward trend was reported in a study in south-western Nigeria [21]. This might be due to the support given to the northern part of the country by development partners in areas of immunization. In addition, we observed annual seasonality of measles, with an increase in the number of cases in the first quarter. Similar patterns have been reported in separate studies conducted in other parts of Nigeria [6, 18, 21].

Furthermore, our study revealed that age less than five years, male sex and failure to receive measles vaccine were associated with measles mortality. Different reports have shown that majority of measles deaths occur in children under the age of five years [1, 24]. As regards male sex having higher mortality than females, this finding might be attributed to the health care seeking behaviors of mothers in the country as it was reported that mothers tend to seek advice/care for their female children than the male counterparts [5].

Our study had some limitations. The data was not collected primarily for this purpose and was incomplete. Thus, only cases with complete information on at least 3 variables were included in the study. Also, not all suspected measles cases get notified and reported through the surveillance system. This may have under-estimated the number of measles cases and the vaccination coverage reported.

## Conclusion

Measles remains a public health concern in Jigawa State. Case-based surveillance provided an insight into understanding the epidemiology of measles infection in Jigawa State. There was poor vaccination coverage among cases and laboratory investigation was low. Compared to those who had received at least one dose of measles vaccine, those who had never been vaccinated were more likely to die. The government of Nigeria through NCDC should strengthen laboratory testing capacity and Jigawa state government should revamp routine immunization and ensure every eligible child is reached during Routine Immunization and Supplemental Immunization Activities to build herd immunity and interrupt measles transmission in Jigawa state.

### What is known about this topic

- Measles is a highly contagious vaccine preventable viral disease targeted for elimination by the year 2020;
- Despite decrease in global measles deaths, measles is still common in many developing countries, particularly in Africa and Asia.

### What this study adds

- There is a decreasing trend and seasonal variation in measles cases in Jigawa State, Nigeria;
- Measles mortality was associated with age, sex and vaccination status.

## Competing interests

The authors declare no competing interests.
